# Bazedoxifene as a novel GP130 inhibitor for Colon Cancer therapy

**DOI:** 10.1186/s13046-019-1072-8

**Published:** 2019-02-08

**Authors:** Jia Wei, Ling Ma, Yi-Hui Lai, Ruijie Zhang, Huameng Li, Chenglong Li, Jiayuh Lin

**Affiliations:** 1 Department of Hematology, Tongji Hospital, Tongji Medical College, Huazhong University of Science and Technology, Wuhan, 430030 People’s Republic of China; 2 Department of Biochemistry and Molecular Biology, University of Maryland School of Medicine, 108 N. Greene Street, Baltimore, MD 21201 USA; 3 33 Linsen Road, Chungshan District, Taipei, Taiwan; 4 Biophysics Graduate Program, The Ohio State University, Columbus, OH 43210 USA; 5 College of Pharmacy, University of Florida, Gainesville, FL 32610 USA

**Keywords:** Colon cancer, Bazedoxifene, Oxaliplatin, GP130, IL-11, STAT3

## Abstract

**Background:**

Interleukin-11 (IL-11), a dominant IL-6 family cytokine, is involved in tumorigenesis, tumor progression and differentiation in colon cancer cells. IL-11 signaling has been recently identified as a potential therapeutic target in colon cancer. Bazedoxifene, a third- generation selective estrogen modulator approved by the Food and Drug Administration (FDA), is a novel inhibitor of IL-11/GP130 signaling discovered by docking modeling.

**Methods:**

In this study, the inhibition efficacy of bazedoxifene in colon cancer cells and its potential mechanism were investigated in vitro and in vivo by using MTT cell viability assay, BrdU cell proliferation assay, colony formation assay, wound-healing/cell migration assay, immunofluorescence, western blot assay and the mouse xenograft tumor model.

**Results:**

Bazedoxifene inhibits phosphorylation of signal transducer and activator of transcription 3 (p-STAT3) and its nuclear translocation induced by IL-11 in colon cancer cells. It also inhibits p-STAT3 induced by IL-6 and IL-11 but not by OSM or STAT1 phosphorylation induced by INF-γ in human colon cancer cells. In addition, bazedoxifene can significantly inhibit phosphorylation of AKT and STAT3 downstream targets. Furthermore, bazedoxifene alone or together with oxaliplatin can significantly induce apoptosis, inhibit cell viability, cell colony formation and cell migration in colon cancer cells. Knock-down of IL-11R can reduce the sensitivity of colon cancer cells to bazedoxifene. IL-11 can reduce the efficacy of oxaliplatin-mediated inhibition of cell viability. Consistent with in vitro findings, bazedoxifene alone also attenuated HCT-15 xenograft tumor burden and reduced p-STAT3, p-AKT and p-ERK in vivo*.* Its combination with oxaliplatin attenuated DLD-1 xenograft tumor burden and reduced p-STAT3 in vivo*.*

**Conclusions:**

Taken together, these results support bazedoxifene as a novel and effective therapeutic agent targeting IL-11/GP130 signaling for human colorectal cancer therapy.

**Electronic supplementary material:**

The online version of this article (10.1186/s13046-019-1072-8) contains supplementary material, which is available to authorized users.

## Background

Colorectal cancer (CRC) is the third most common cancer worldwide and the second leading cause of cancer-related deaths in the United States [1]. Its occurrence is driven by the accumulation of genetic mutations affecting more than one molecular pathway [2]. Its growth and survival are modulated by cytokine-mediated activation [3]. Therapies available for treatment of colon cancer include surgery, chemotherapy, radiation therapy, immunomodulatory therapy and molecule targeted treatment [4–6]. Despite impressive accomplishments, the five-year survival rate remains less than 10% for metastatic colon cancer. Nearly all metastatic colorectal cancer patients eventually become resistant to oxaliplatin with a median time to progression of 8.7 months [7, 8]. Novel targeted drugs are desired to make an improvement in the therapy of this disease.

STAT3 is a crucial and well-known mediator of malignant progression in CRC [9, 10]. An ever-increasing number of reports correlate excessive GP130/STAT3 signaling with the progression of colon cancer [11]. Once activated, it plays a critical role in the oncogenesis, proliferation, metastasis and invasion of colon cancers by up-regulating the expression of downstream genes, including cyclin D1, c-myc, bcl-XL, survivin etc. Thus far, a series of STAT3-activating cytokines that promote colon cancer has been identified [3]. IL-11, a member of the IL-6 family of cytokines, has been recently identified as potentially the most important cytokine in promoting colon cancer through exclusive utilization of GP130 homodimers when bound to its receptor, IL-11R [12]. The interleukin (IL)-6 family of cytokines comprises 10 members: IL-6, IL-11, ciliary neurotrophic factor (CNTF), cardiotrophin-1 (CT-1), cardiotrophin-like cytokine (CLC), leukemia inhibitory factor (LIF), neuropoietin (NP), oncostatin M (OSM), IL-27, and IL-31 [13]. IL-11 and IL-6 are the only known cytokines that initiate signal transduction via a GP130 homodimer; all other cytokines utilize heterodimers of GP130 in combination with a second signal-transducing receptor [14]. In classic signaling, IL-11 binds to the membrane-bound IL-11R to initiate signal transduction. Recently, it was found that IL-11 signaling can also be initiated via soluble IL-11R, known as trans-signaling [15]. Upon the formation of IL-11/IL-11R/GP130 hexameric complex, IL-11 mainly mediated cancer development through the induction and activation of the JAK/STAT3 signaling pathway [16–18]. It has already been found to contribute to tumorigenesis in several types of solid malignancies [19, 20]. In colon cancer, the up-regulated IL-11 and IL-11R were also found to be highly expressed in samples of CRC patients [21]. The dominance of IL-11 over IL-6 as the cytokine enabling tumor outgrowth from the gastrointestinal epithelium also extends into clinically more prevalent situations that occur independently of overt inflammation and/or colitis. Accordingly, low IL-11 levels correlate with reduced resistance toward chemotherapy of some solid cancers. Several studies have emphasized the potential tumorigenic role of IL-11 in colon cancer as well as its potential role as a target in colon cancers [3, 22]. IL-11 has a stronger correlation with elevated STAT3 activation in human gastrointestinal cancers. All these findings demonstrate that therapeutic inhibition of IL-11/GP130 signaling can be used for the treatment of gastrointestinal cancers.

The FDA-approved drug bazedoxifene, known as a selective estrogen modulator, is currently used for the postmenopausal osteoporosis [23]. Recently, using multiple-ligand simultaneous docking and drug repositioning, we identified bazedoxifene as a novel small-molecule inhibitor of GP130 [24]. Our previous work has repositioned this drug as a potent GP130 inhibitor in pancreatic cancer therapy [25], but its effects on colon cancer have not been investigated. Although IL-6 has been associated with many epithelial cancers, IL-11 acts as a more potent driver of colorectal cancers [22]. Since activated STAT3 and IL-11 were found to be overexpressed and promote tumorigenesis in most colon cancers [21, 26, 27], we hypothesized a potential inhibitory role of bazedoxifene in colon cancers. In this study, bazedoxifene was investigated either as a single agent or in combination with oxaliplatin in colon cancer cells. Oxaliplatin is the third-generation of platinum drugs which form platinum-DNA adducts to block DNA replication, leading to cell death and cell cycle arrest [28]. It has been widely used in the first-line chemotherapy in colon cancer. However, nearly a half of the patients receiving oxaliplatin still develop resistance and its action mechanism has not been fully clarified. Due to the increasing number of examples of oxaliplatin resistances, new strategies to overcome this pitfall are desired.

In this study, we investigated the anti-cancer effect of bazedoxifene on human colon cancer cells in vitro and in vivo by blocking the IL-11/GP130 pathway. We also demonstrated the efficacy of the combination therapy of bazedoxifene and oxaliplatin in human colon cancer cells. Our results may provide a novel approach to the molecule-targeted treatment in colon carcinoma.

## Methods

### Cell lines and reagents

Human colon cancer cell lines (DLD-1, HCT-15, and HCT-116) were purchased from ATCC (the American Type Culture Collection, Manassas, VA, USA). They were cultured in Eagle’s Minimum Essential Medium (DMEM supplemented with fetal bovine serum (FBS) and 1% penicillin/streptomycin. Cells were cultured for less than 3 months before reinitiating cultures. All cell lines were cultured in a humidified 37°C incubator with 5% CO_2_.

Bazedoxifene was purchased from Acesys Pharmatech (USA), and oxaliplatin was purchased from LC Laboratories (Woburn, MA, USA). All drugs were dissolved in sterile dimethyl sulfoxide (DMSO) to make 20 mmol/l (mM) stock solutions. IL-6, IL-11, OSM and IFN-γ were purchased from Cell Signaling. Neutralized human GP130 antibody, neutralized IL-11 antibody and control IgG antibody were purchased from R&D Systems™ (Minneapolis, USA). The powders were dissolved in sterile PBS to make 100 ng/μl stock solutions. IL-11 and BrdU (bromodeoxyuridine) Cell Proliferation Assay Kit were purchased from Cell Signaling (Beverly, MA, USA). The powder was dissolved in sterile PBS to make a 100 ng/μl stock solution. Aliquots of the stock solution were stored at − 20°C.

### MTT cell viability assay

Human colon cancer cell lines (DLD-1, HCT-15, and HCT-116) were seeded in 96-well plates at a density of 3000 cells per well. The next day, cells were treated as indicated and incubated at 37°C for a period of 24–72 h. Then, 25 μl of 3-(4,5-dimethylthiazolyl)-2,5-diphenyltetrazolium bromide (MTT, Sigma, USA) was added to each sample in a volume of 100 μl and incubated for 4 h. After that, 150 μl of N, N-dimethylformamide (Sigma, USA) solubilization solution was added to each well to dissolve the formazan. The absorbance was read at a wavelength of 595 nm. A combination index (CI) was dertermined using the data obtained from MTT assay with CompuSyn software [29]. CI values indicate a synergistic effect when < 1, an antagonistic effect when > 1, and an additive effect when equal to 1. Half-maximal inhibitory concentrations (IC_50_) were determined by the GraphPad Prism software 7.0 (USA).

### Caspase-3/7 activity

Cells were cultured in the respective media and treated with bazedoxifene and oxaliplatin alone or in combination as described above. Caspase-3/7 activity was measured using the Caspase-3/7 Fluorescence Assay kit (Cayman, Ann Arbor, MI, USA) according to the manufacturer’s instruction. The fluorescence intensity of each well was read using excitation at 485 nm and emission at 535 nm.

### BrdU cell proliferation assay

The proliferative activities intrinsic to DLD-1, HCT-15, and HCT-116 colon cancer cell lines were assessed by BrdU (bromodeoxyuridine) incorporation assay. Briefly, cells were seeded in 96-well plates in quadruplicate at a density of 5000 cells per well in the routine growth medium for 24 h. The next day, cells were grown in the same routine medium but devoid of FBS for another 24 h. Such a serum-depleted growth condition was continued throughout the assay. Twenty-four hours after the growth induction, cells were further cultivated with BrdU reagent for 1 h, and the ones that incorporated BrdU into proliferating DNA were quantified as described in the manufacturer’s instructions.

### siRNA transfection

Colon cancer cell lines (DLD-1, HCT-15, HCT-116) were transfected with 10 nM of negative control siRNA or human IL-11Rα siRNA (Santa Cruz, USA) using Lipofectamine 2000 (Invitrogen, USA) according to the manufacturer’s instructions. After 48 h of transfection, cells were harvested and lysed for western blot or processed for MTT cell viability assay. Cells were then treated with bazedoxifene for another 48 h. Cell viability was then analyzed by MTT assay.

### Colony formation assay

Human colon cancer cells (DLD-1, HCT-15, and HCT-116) were grown in six-well cell culture plates and treated with bazedoxifene and oxaliplatin at the indicated doses. After trypsinization, the viable cells were collected and seeded at 1000 cells per well in 6-well plates and allowed to grow until DMSO-treated control cells reached confluence for two to three weeks. Cells were washed with PBS twice and fixed with cold methanol for 30 min followed by staining with 1% crystal violet dye in 25% methanol at room temperature for 2 h. The plates were then rinsed with distilled water and dried prior to scanning.

### Wound-healing/cell migration assay

When colon cancer cells (DLD-1, HCT-15, and HCT-116) were 100% confluent, the monolayer was scratched to the same width using a yellow pipette tip. After washing, these cells were then treated with bazedoxifene alone or in combination with oxaliplatin at indicated concentrations. After a 24 to 72-h culture, when the wound in the DMSO control group was completely healed, images were captured by inverted microscope (Nikon, Eclipse TS100, Japan). The percentage of wound-healing was measured by the ImageJ software (National Institutes of Health, USA) and calculated by the equation: percent wound healing = average of (gap area before treatment - gap area after treatment)/gap area before treatment.

### Immunofluorescence

DLD-1 cells were seeded on glass coverslips in a 6-well plate. The next day, the cells were cultured in serum-free medium for 24 h and pre-treated with bazedoxifene (10 μM) for 2 h, followed by induction with 50 ng/mL IL-11 for 30 min. Cells were fixed with cold methanol for 15 min and blocked with 5% normal goat serum and 0.3% Triton X-100 in PBS for 1 h. The cells were incubated with primary antibodies against p-STAT3^Y705^ (Cell Signaling, 1:100) overnight at 4°C. After incubation with anti-rabbit FIFC-conjugated secondary antibody (Invitrogen, 1: 200), the cells were mounted with Vectashield Hardset mounting medium with DAPI (Vector Laboratories, Burlingame, CA, USA). Photomicrographs were captured by Leica Microsystems (Bannockburn, IL, USA).

### Western blot (WB) assay

Human colon cancer cell lines (DLD-1, HCT-15, and HCT-116) at 50–60% confluence were harvested after an overnight treatment with bazedoxifene or DMSO, and then lysed in cold RIPA lysis buffer containing protease inhibitor cocktail and phosphatase inhibitor cocktail. The lysates were subjected to 10% or 12% SDS-PAGE gel and transferred to a PVDF membrane. Membranes were probed with a 1:1000 dilution of specific primary antibody and 1:10,000 HRP-conjugated secondary antibody. Primary antibodies against phosphorylated STAT3 (Tyr705, p-STAT3^Y705^), IL-6, BCL-XL, c-MYC, survivin, cyclin D1, STAT3, AKT, ERK, phospho-specific extracellular signal-regulated kinase (ERK) 1/2 (threonine 202/Tyrosine 204), phosphorylated-AKT (Ser473), GAPDH and secondary antibodies were all from Cell Signaling Technology (Beverly, MA, USA). Primary antibodies against IL-11, IL-11Rα and IL-6R were from Abcam (Cambridge, MA, USA). Membranes were analyzed using enhanced chemiluminescence plus reagents and scanned with the Storm Scanner (Amersham Pharmacia Biotech Inc., Piscataway, NJ).

### Mouse xenograft tumor model

All animal studies were conducted in accordance with the principles and standard procedures approved by IACUC of the Research Institute at University of Maryland, Baltimore. HCT-15 cells or DLD-1 cells (1 × 10^7^) with an equal volume of matrigel (BD Science, Franklin Lakes, NJ) were injected subcutaneously into one side of flank area of 6-week-old female athymic nude mice, which were purchased from Harlan (Indianapolis, IN, USA). For the HCT-15 xenograft mouse model, after five days of tumor development, mice were divided into two treatment groups consisting of four mice: DMSO vehicle control group and gavage injection of bazedoxifene (10mg/kg/d) group. For the DLD-1 xenograft mouse model, after five days of tumor development, mice were divided into four treatment groups consisting of five mice: DMSO vehicle control group, gavage injection of bazedoxifene (10 mg/kg/d) group, intraperitoneal injection of oxaliplatin (5 mg/kg, twice a week) or combination injection.

Tumor growth was determined by measuring the length (L) and width (W) of the tumor every other day with a caliper, and tumor volume was calculated by the following formula: volume = 0.52 × LW^2^. After 14 or 16 days of treatment, tumors were harvested, snap-frozen in dry ice, and stored at − 80 °C. Tumors tissue homogenates were lysed and separated by SDS-PAGE to examine the expression of p-STAT3^Y705^, phosphorylated-AKT^Ser473^, phospho-specific extracellular signal-regulated kinase (ERK) 1/2^threonine 202/Tyrosine 204^, STAT3, AKT, ERK and GAPDH.

### Statistics

The significance of correlations was assessed using the GraphPad Prism software 7.0 (GraphPad Software, Inc., USA). Unpaired t-tests were used for analyses assuming Gaussian populations with a 95% confidence interval. Data are presented as the mean ± standard deviation (SD). Differences were analyzed with the Student t-test, and significance was set at *p* < 0.05. *, ** and *** indicates *p* < 0.05, *p* < 0.01 and *p* < 0.001, respectively.

## Results

### Bazedoxifene is identified as a novel inhibitor of IL-11/GP130

Our previous work has shown that bazedoxifene can bind to the GP130 D1 domain. Based on computer modeling, IL-11 binds via site III to the second copy of the GP130 D1 domain in a dimer via the key Trp168 and Leu72 residues. Docking bazedoxifene to the GP130 D1 domain shows that the indole moiety and azepanyl of bazedoxifene effectively compete for IL-11 binding with the Trp168 and Leu72 residues, respectively. Additionally, bazedoxifene forms aromatic and hydrogen bonds with Asn92 and Tyr94 in binding hot spots of GP130 (Additional file 1: Figure S1). This suggests that bazedoxifene should effectively block IL-11/GP130 signaling by disrupting Trp168 and Leu72 binding.

### The IL-11/GP130 pathway is a potential target in human colon cancer cell lines

**Fig. 1:**
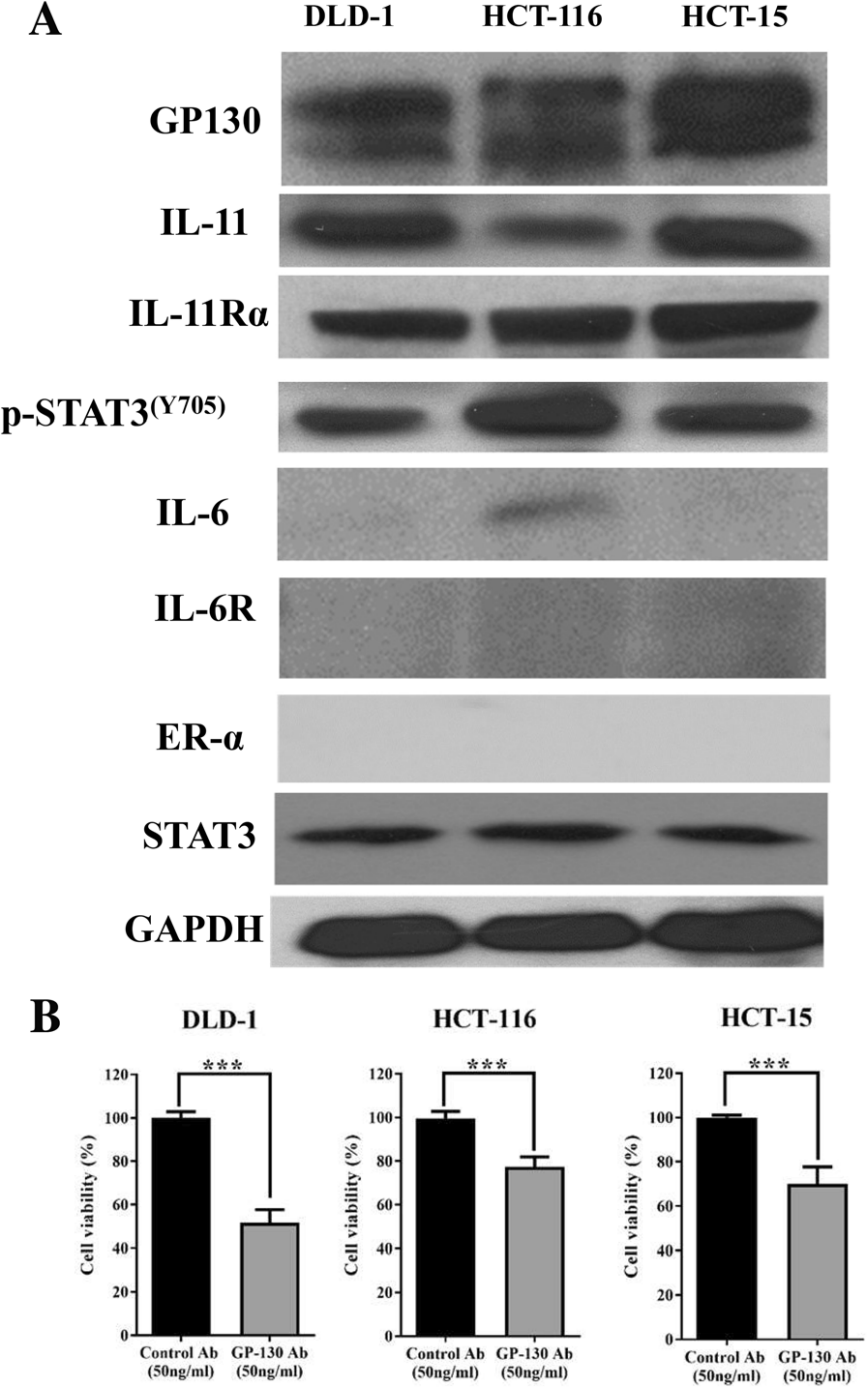
Human colon cancer cells are sensitive to GP130 inhibition. **a**: The expression of GP130, IL-11, IL-11Rα, IL-6, IL-6R, p-STAT3(Y705) and ER-α was evaluated in colon cancer cells. DLD-1, HCT-116, and HCT-15 cells were harvested, and the protein expression was detected by western blot. GAPDH served as a loading control. **b**: 5000 cells per well of DLD-1, HCT-15 and HCT-116 cells were seeded in 96-well plates and treated with neutralized GP130 antibody at 50 ng/ml in 2% FBS medium for 48 h. The IgG antibody at the same concentration served as a control. Cell viability was measured by MTT assay. (***, *p* < 0.001)

The expression of GP130, IL-11, IL-11 receptor α (IL-11Rα), IL-6, IL-6 receptor (IL-6R) and STAT3 activation were examined by western blot in DLD-1, HCT-15, and HCT-116 colon cancer cell lines. As indicated in Fig. 1a, all three colon cancer cells highly expressed GP130, IL-11, IL-11Rα and p-STAT3. They expressed IL-6 at a low level. Estrogen receptor α (ER-α) and IL-6R could not be detected in these three colon cancer cells. When blocking GP130 signaling by the human neutralized GP130 antibody, the cell viability of three colon cancer cell lines was significantly reduced (Fig. 1b). These results indicated that the IL-11/GP130 pathway could be a potential target for treatment of colon cancer.

### Bazedoxifene inhibits colon cancer cell colony-forming and cell migration ability in vitro

**Fig. 2:**
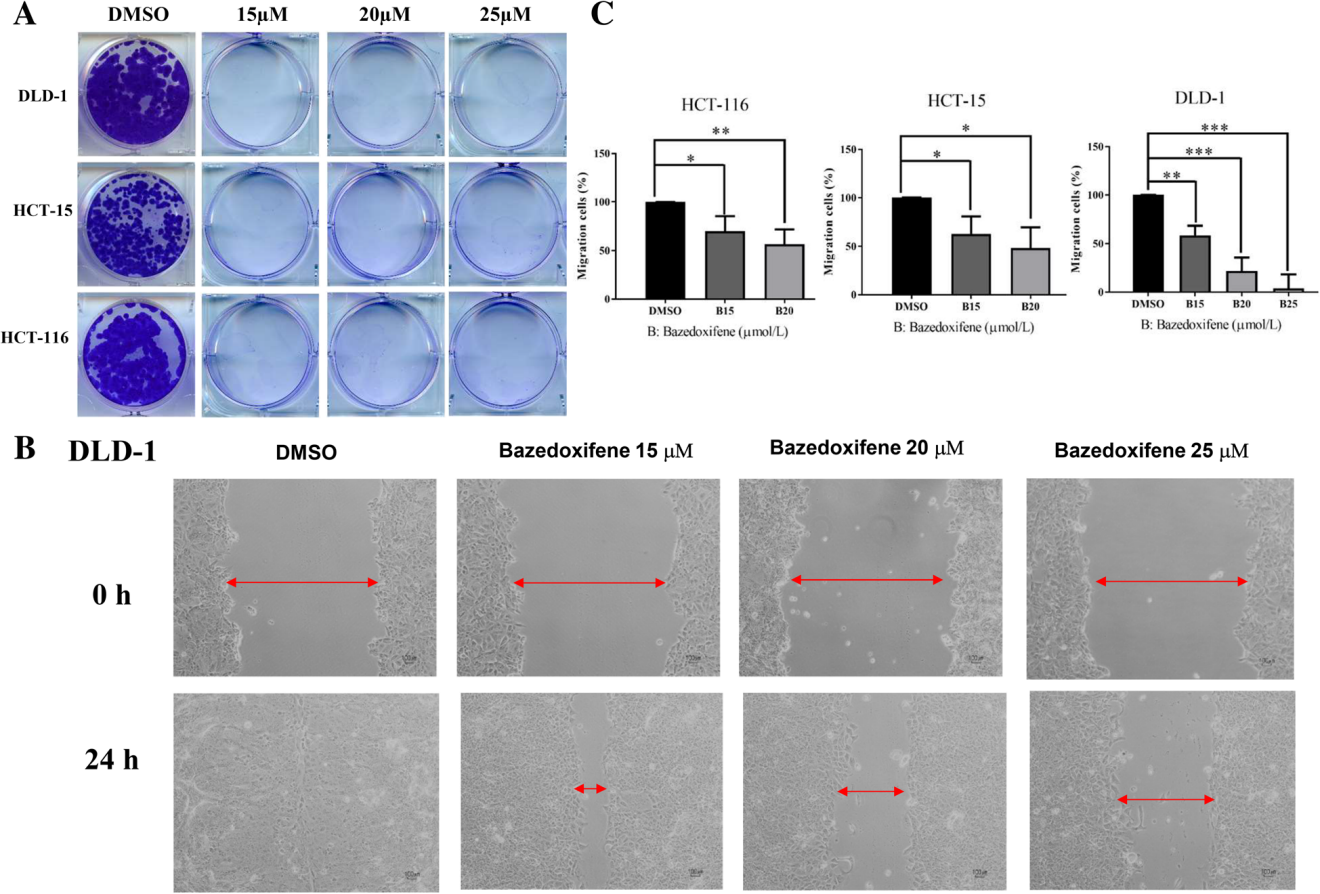
Bazedoxifene inhibits colony formation and cell migration in human colon cancer cells. **a**: Colony formation assay was conducted in DLD-1, HCT-15, and HCT-116 cells as described in materials and methods. DLD-1, HCT-15, and HCT-116 cells were treated with bazedoxifene at indicated concentrations for 24 h, re-seeded at 1000 cells per well and cultured for 2–3 weeks to grow clones. **b**: A representative picture shows DLD-1 cells in a wound-healing assay. It was conducted by scratching the cells with a yellow pipette tip when DLD-1 cells grew into a monolayer. Then, cells were treated with bazedoxifene (15, 20 and/or 25 μM) and allowed to migrate into the scratched area for 24 h. Red arrows indicate a gap in the scratched area. **c**: The percentage of migrating area in wound-healing assay was quantified in DLD-1, HCT-15, and HCT-116 cells. (*, *p* < 0.05; **, *p* < 0.01; ***, *p* < 0.001)

We first tested the inhibitory effect of bazedoxifene on DLD-1, HCT-15, and HCT-116 colon cancer cells. The IC50 for DLD-1, HCT-15, and HCT-116 colon cancer cells were 8.70 ± 0.18 μmol/l, 6.25 ± 0.16 μmol/l and 9.02 ± 0.97 μmol/l, respectively. Since cell colony formation and cell migration are likely two important processes in colon cancer tumorigenesis and metastasis, the colony formation and wound-healing assays were performed. As shown in Fig. 2a, colon cancer cells showed a prominent decrease in their ability to recover and form colonies after bazedoxifene treatment. Since activation of GP130/STAT3 is involved in cell migration, the wound-healing assay was performed on DLD-1, HCT-15, and HCT-116 cells. As shown in Fig. 2b-c, bazedoxifene treatment resulted in a dose-dependent decreased migration ability of colon cancer cells.

### Bazedoxifene potentiates the anti-tumor activity through the IL-11/GP130 /STAT3 pathway

**Fig. 3:**
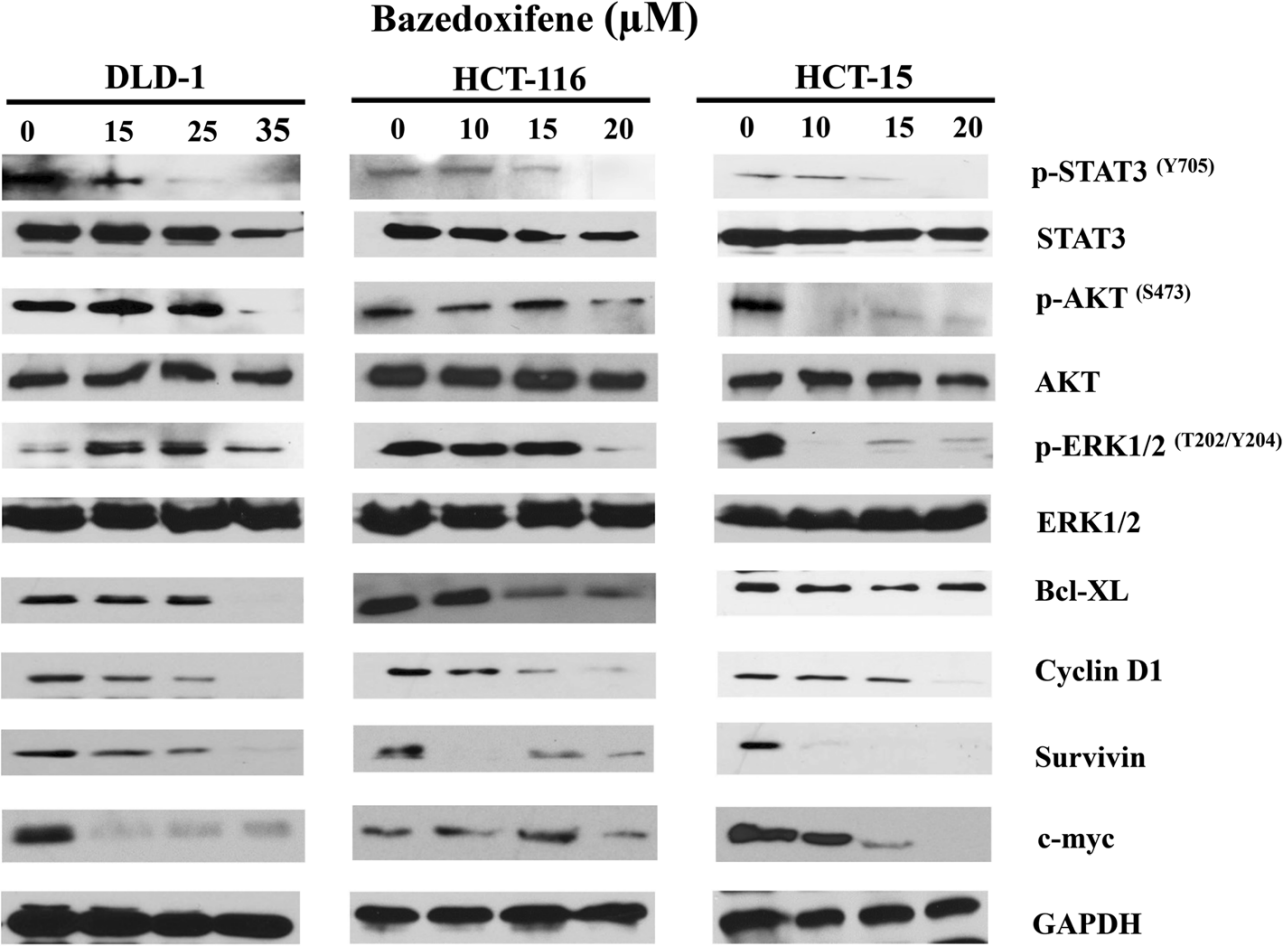
Bazedoxifene inhibits STAT3, AKT and ERK phosphorylation and downregulates expression of STAT3 downstream proteins. DLD-1, HCT-15, and HCT-116 human colon cancer cells were treated with different concentrations of bazedoxifene (10~35 μM) for 24 to 48 h. The protein expression of p-STAT3^Y705^, p-AKT^S473^, p-ERK1/2^T202/Y204^, BCL-XL, cyclin D1, survivin, c-myc, ERK, AKT and STAT3 were detected by western blots with GAPDH as a loading control

Since activation of STAT3 phosphorylation can be detected in DLD-1, HCT-15 and HCT-116 cells, we tested whether bazedoxifene can inhibit the activation of STAT3. The results (Fig. 3) showed that bazedoxifene reduced the levels of phosphorylated-STAT3^Y705^ in a dose-dependent manner in all three colon cancer cells. Phosphorylated-AKT (p-AKT) was also downregulated by bazedoxifene in all three colon cancer cells. Phosphorylated-ERK (p-ERK) was downregulated by bazedoxifene in HCT-15 and HCT-116 but not in DLD-1 cells. The proteins downstream of the STAT3 pathway, including cyclin D1, survivin and c-myc, also decreased correspondingly in all three colon cancer cell lines. Bcl-XL was decreased in DLD-1 and HCT-116 cells.

**Fig. 4:**
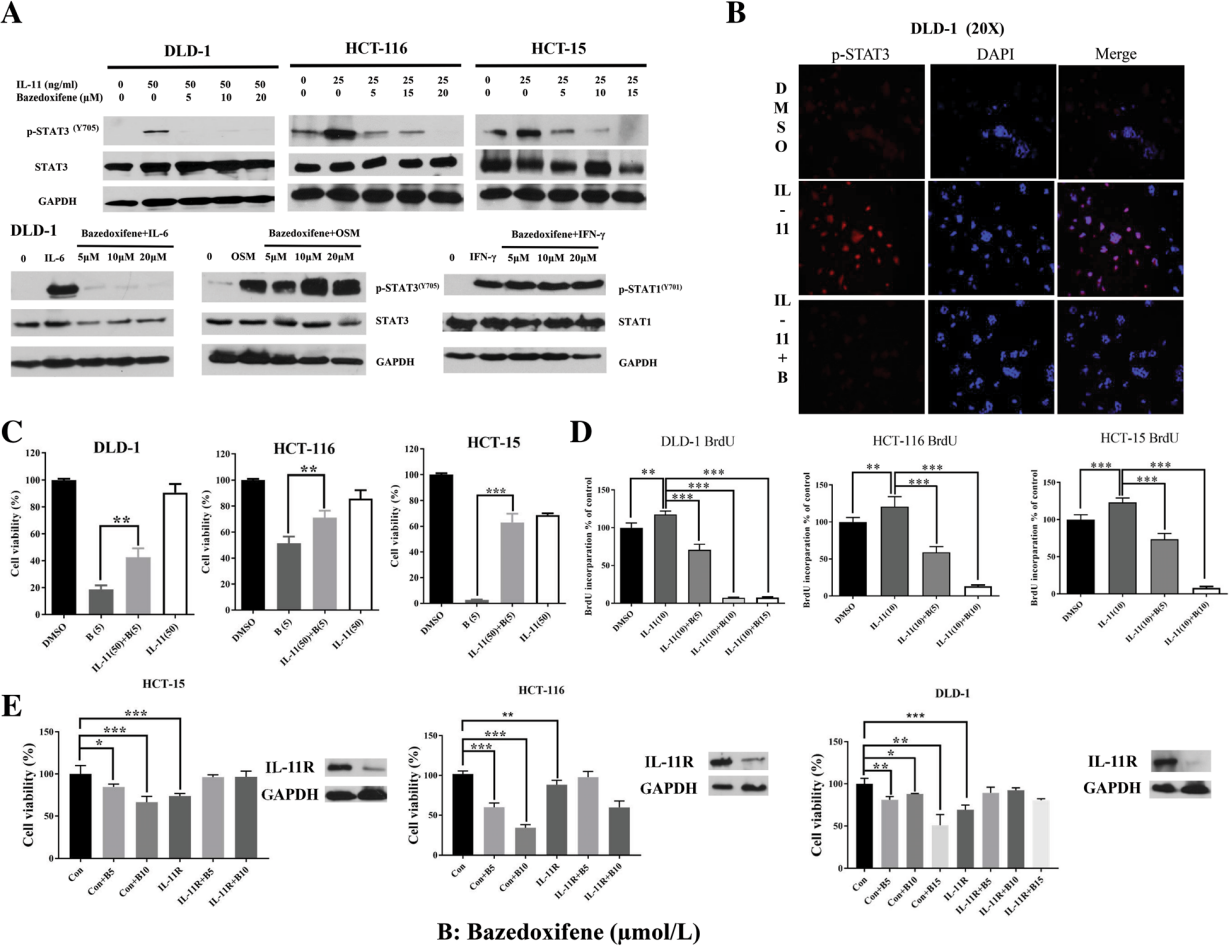
Bazedoxifene inhibits induction of STAT3 phosphorylation and cell proliferation by IL-11. **a**: DLD-1, HCT-116, and HCT-15 cells were starved in serum-free medium for 24 h and pre-treated with bazedoxifene (5~20 μM) for 2 h. Then, 50 ng/ml (DLD-1 cells) or 25 ng/ml IL-11 (HCT-116 and HCT-15 cells), 50 ng/ml OSM (DLD-1 cells) and 50 ng/ml IFN-γ (DLD-1 cells) were added for stimulation. The p-STAT3^Y705^, p-STAT1^Y701^, STAT3, STAT1 and GAPDH were assessed by western blot analysis. **b**: DLD-1 cells were starved in serum-free medium for 24 h and pre-treated with bazedoxifene (10 μM) for 2 h followed by IL-11 stimulation (50 ng/ml). STAT3 nuclear translocation was detected by immunofluorescence. **c**: 3000 cells per well of DLD-1, HCT-116, and HCT-15 were seeded in 96-well plates and starved in serum-free medium for 24 h. The next day, cells were pretreated with 5 μM bazedoxifene for 2 h alone or followed with 50 ng/ml IL-11 stimulation. After 24-h treatment, cell viability was determined by MTT assay. **d**: Induction of cell proliferation and p-STAT3 is inhibited by bazedoxifene in colon cancer cells. 8000 cells per well of DLD-1, HCT-116 and HCT-15 cells were seeded in 96-well plates and then starved in serum-free medium for 24 h. Pretreatment with 5~15 μM bazedoxifene for 4 h was followed by 10 ng/ml IL-11 stimulation for 24 h. BrdU assay was performed as described previously. **e**: IL-11Rα is knocked down in DLD-1, HCT-116, and HCT-15 cells. Cells were transfected with 10 nM of negative control siRNA or human IL-11Rα siRNA. After 48 h, cells were harvested and lysed for western blot or processed for MTT cell viability assay. Cells were then treated with bazedoxifene for another 72 h. IL-11R was assessed by western blot analysis with GAPDH as a control. Cell viability was determined by MTT assay. (*, *p* < 0.05; **, *p* < 0.01; ***, *p* < 0.001)

Since repositioning identified a novel function of bazedoxifene against interaction between IL-11 and GP130, we tested whether the inhibition mechanism was through targeting IL-11 in DLD-1 HCT-15, and HCT 116 colon cancer cells. The WB results showed that IL-11 can induce the p-STAT3 expression in all three colon cancer cells, and this activation can be reversed by bazedoxifene (Fig. 4a). It also inhibits p-STAT3 induced by IL-6 and IL-11 but not by OSM or STAT1 phosphorylation induced by INF-γ in DLD-1 cells (Fig. 4a). Immunofluorescence results showed that STAT3 nuclear translocation induced by IL-11 is inhibited by bazedoxifene in DLD-1 cells (Fig. 4b). The MTT array showed that colon cancer cells inhibited by bazedoxifene could be partially rescued by excessive administration of IL-11 (Fig. 4c). When treated with IL-11 (10 ng/ml), the cell proliferation ability of three colon cancer cells was induced. The induced cell proliferation was inhibited by bazedoxifene in colon cancer cells (Fig. 4d). To further determine the role of the IL-11/GP130/STAT3 pathway inhibition in colon cancer treatment with bazedoxifene, DLD-1, HCT-116 and HCT-15 cells were transfected with IL-11R siRNA for 48 h and treated with bazedoxifene for another 24 h. The knock-down of IL-11R was confirmed by western blot. As shown in Fig. 4e, the level of IL-11R was decreased. Cell viability was significantly reduced in the control siRNA group but was not reduced in IL-11R siRNA group at the same bazedoxifene concentration. All these results confirmed that bazedoxifene exerted its inhibitory role in colon cancer cells through inhibition of the IL-11/GP130/STAT3 signaling pathway.

### Bazedoxifene enhances the efficacy of oxaliplatin by blocking IL-11

**Fig. 5:**
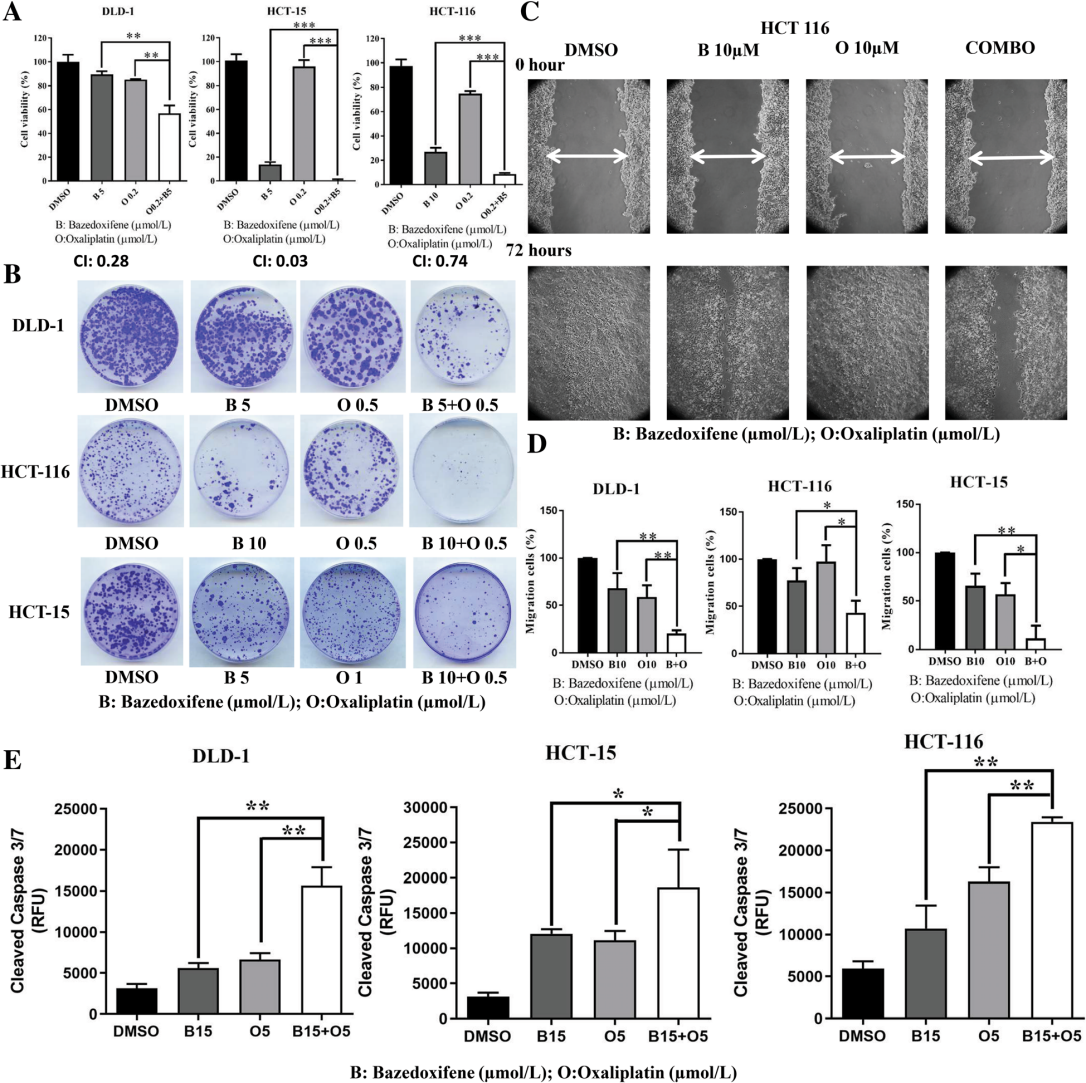
Bazedoxifene shows synergistic effects in combination with oxaliplatin. **a**: DLD-1, HCT-15, and HCT-116 cells were seeded in 96-well plates at a density of 3000 cells per well, incubated overnight and then treated with bazedoxifene, oxaliplatin or bazedoxifene and oxaliplatin at the indicated doses for 72 h. Cell viability was determined by MTT assay. The CI values of all combination treatments were calculated by CompuSyn software. **b**: DLD-1, HCT-15, and HCT-116 cells were treated with bazedoxifene, oxaliplatin or bazedoxifene and oxaliplatin combination at the indicated doses for 72 h. After treatment, the same numbers of cells were seeded and cultured in a drug-free medium for 1–2 weeks. Colonies were fixed by ice-cold methanol and stained with 1% crystal violet. **c**: A representative picture shows HCT-116 cells in a wound-healing assay. It was conducted by scratching the cells with a yellow pipette tip when HCT-116 cells grew into a monolayer. Then, cells were treated with 10 μM bazedoxifene, 10 μM oxaliplatin and 10 μM bazedoxifene with 10 μM oxaliplatin in combination. Cells were allowed to migrate into the scratched area for 24 h (DLD-1 and HCT-15 cells) or 72 h (HCT-116 cells). The white arrows indicate the gap in scratched area. D: The percentage of migrating area in wound-healing assay was quantified in HCT-116, DLD-1 and HCT-15 cells. E: Eight thousand cells per well of DLD-1, HCT-15, and HCT-116 cells were treated with 15 μM bazedoxifene, 5 μM oxaliplatin and a combination of 15 μM bazedoxifene with 5 μM oxaliplatin for 4 h. The level of cleaved caspase-3/7 (RFU) was measured using Caspase-3/7 Fluorescence Assay kit. (*, *p* < 0.05; **, *p* < 0.01; ***, *p* < 0.001)

To evaluate the synergistic effect of IL-11/GP130 suppression with conventional chemotherapeutic drugs, oxaliplatin, which was approved by FDA for the treatment of colon cancer, was selected in combination with bazedoxifene. The IC50 values of oxaliplatin alone for DLD-1, HCT-15, and HCT-116 colon cancer cells were 17.8 ± 3.58 μmol/l, 3,47 ± 0.76 μmol/l and 14.06 ± 2.64 μmol/l, respectively. The cell viability was significantly reduced by bazedoxifene and oxaliplatin combination treatment compared with either drug alone. All CI values were less than one, indicating synergy between two agents (Fig. 5a). In addition, bazedoxifene plus oxaliplatin significantly reduced the colon cancer cell migration as well as colony formation ability compared with either drug alone (Fig. 5b-d). We then investigated the induction of apoptosis by bazedoxifene alone and in combination with oxaliplatin in colon cancer cell lines. The results showed that the level of cleaved caspase-3/7 was also elevated with drug combination compared with levels for either drug alone (Fig. 5e).

**Fig. 6:**
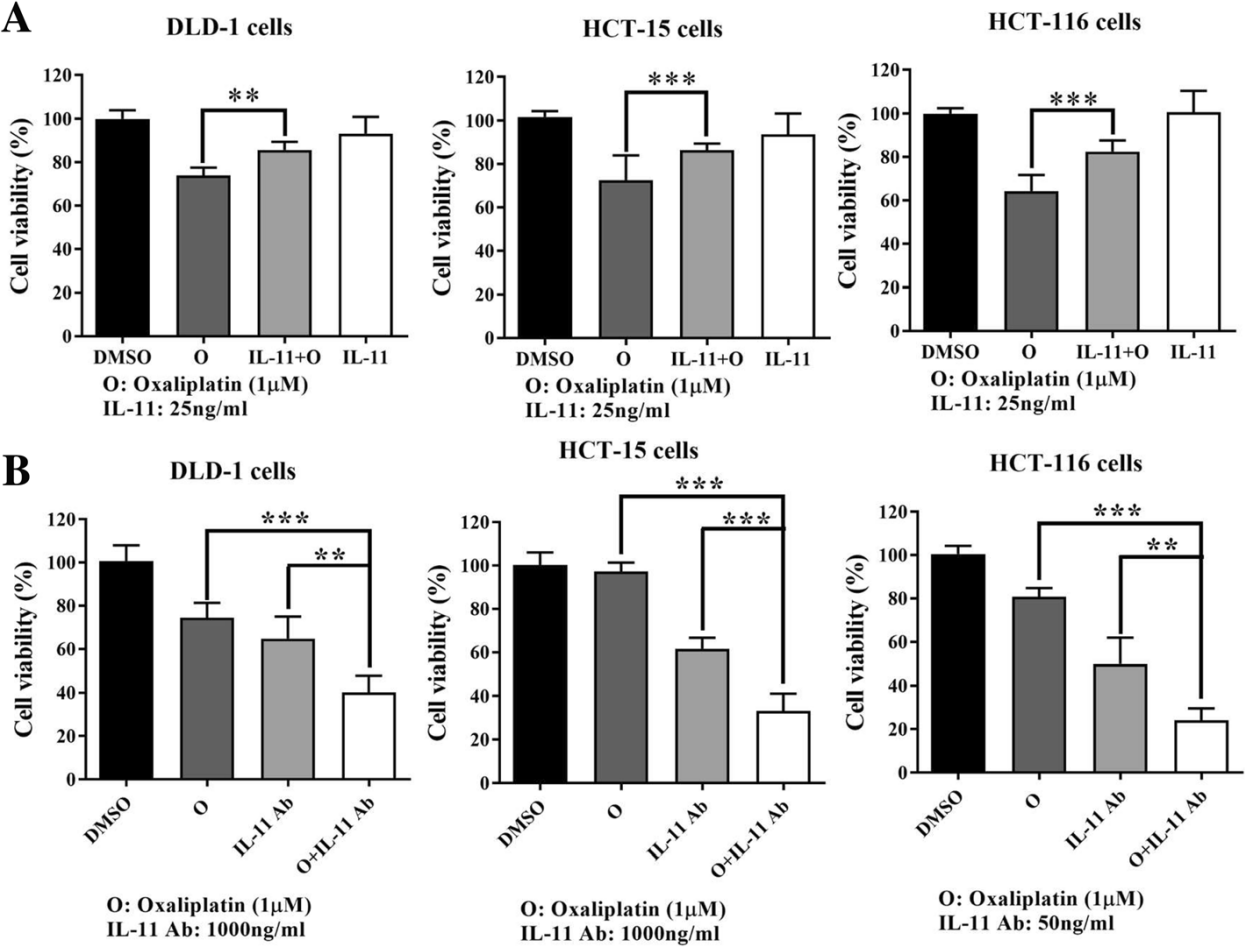
Oxaliplatin resistance mediated by IL-11 can be overcome by blocking the IL-11 pathway. **a**: IL-11 partially rescued the colon cancer cells treated with oxaliplatin. Human colon cancer cells (DLD-1, HCT-15, and HCT-116) were starved in serum-free medium in 96-well plates at a density of 3000 cells per well for 24 h. The next day, the cells were pre-treated with 10 μM oxaliplatin for 2 h followed by 50 ng/ml IL-11 stimulation. After 24 h (DLD-1 cells) or 48 h (HCT-15 and HCT-116 cells) of treatment, cell viability was measured by MTT assay. **b**: The neutralized IL-11 Ab can enhance the efficacy of oxaliplatin. Human colon cancer cells (DLD-1, HCT-15, and HCT-116) were starved in serum-free medium in 96-well plates at a density of 3000 cells per well for 24 h. Neutralized IL-11 antibodies at 1000 ng/ml (DLD-1 and HCT-15 cells) or 50 ng/ml (HCT-116 cells) were added alone or with 1 μM oxaliplatin, which was added 4 h later. After 48-h treatment, cell viability was measured by MTT assay. (**, *p* < 0.01; ***, *p* < 0.001)

We then investigated whether excessive IL-11 could induce drug resistance to oxaliplatin, and the results showed that an addition of IL-11 partially reversed the efficacy of oxaliplatin-mediated inhibition of cell viability in colon cancer cells (Fig. 6a). In addition, MTT assay showed that blocking IL-11 by neutralized IL-11 antibody (Fig. 6b) further enhanced the efficacy of oxaliplatin. These results have suggested that one possible mechanism of oxaliplatin resistance involves IL-11, and our results showed that bazedoxifene could enhance the efficacy of oxaliplatin by inhibiting IL-11 signaling.

### Bazedoxifene inhibited HCT-15 and DLD-1 tumor growth in vivo

**Fig. 7:**
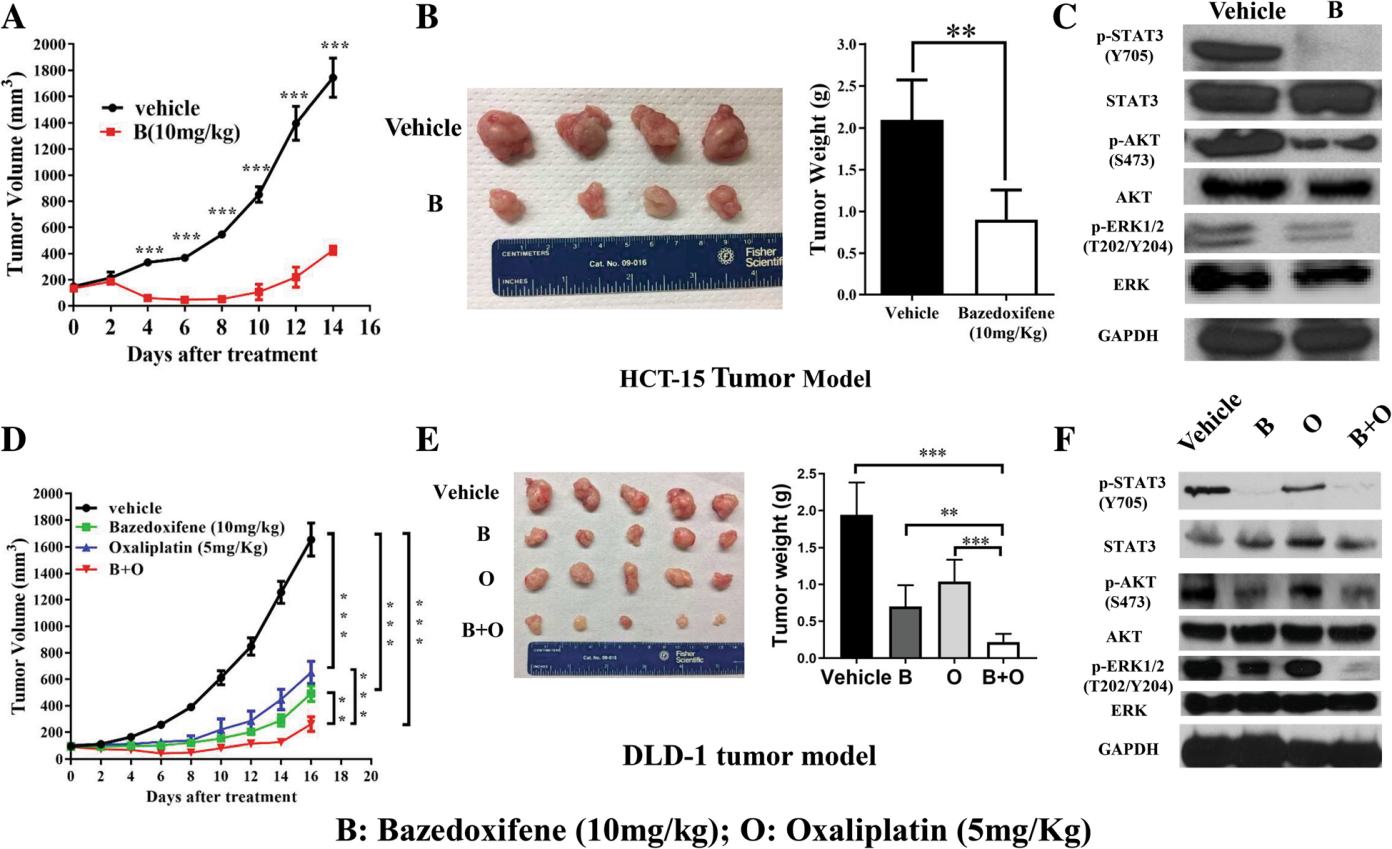
Bazedoxifene inhibited HCT-15 and DLD-1 tumor growth in vivo*.* HCT-15 cells (1 × 10^7^) were injected subcutaneously into nude mice with an equal volume of matrigel. When palpable tumors had formed 5 days later, vehicle or 10 mg/kg bazedoxifene was orally gavaged daily. **a**: Tumor volumes were calculated from serial caliper measurements. **b**: After two weeks of treatment, all mice were euthanized, the tumor mass was resected, and the total mass of each tumor was determined at autopsy (*n* = 4 mice per treatment group). **c**: p-STAT3, STAT3, p-AKT, AKT, p-ERK and ERK were determined using western blot analysis of the harvested tumor tissue. GAPDH served as a loading control. DLD-1 cells (1 × 10^7^) were injected subcutaneously into nude mice with an equal volume of matrigel. When palpable tumors had formed 5 days later, vehicle, 10 mg/kg bazedoxifene, 5 mg/kg oxaliplatin or their combination were orally gavaged daily. **d**: Tumor volumes were calculated from serial caliper measurements. **e**: After two weeks of treatment, all mice were euthanized. The tumor mass was resected, and the total mass of the individual tumor was determined at autopsy (*n* = 5 mice per treatment group). F: The phosphorylation level of STAT3, AKT and ERK was determined using western blot analysis of the harvested tumor tissue. GAPDH served as a loading control. (**, *p* < 0.01; ***, *p* < 0.001)

To address the question whether bazedoxifene can inhibit colon tumor growth in vivo, a xenograft tumor nude mice model was used. For the HCT-15 xenograft model, with 10 mg/kg bazedoxifene treatment, tumor volume and resected tumor weight were significantly reduced compared with the vehicle group (Fig. 7a-b). Further, the resected tumor tissues were examined by WB. The results showed that bazedoxifene reduced the expression of p-STAT3 ^Y705^, p-AKT^S473^ and p-ERK1/2^T202/Y204^, which is consistent with an in vitro study (Fig. 7c). Then, we tested whether the combination of bazedoxifene and oxaliplatin had stronger inhibitory effects than single-drug treatment on DLD-1 xenograft tumor growth. The results showed that the combination of bazedoxifene and oxaliplatin dramatically decreased tumor growth compared to both vehicle and monotherapy treatments (Fig. 7d). The tumor weight of resected tumor mass was lower in the combination treatment group compared to both vehicle and monotherapy groups (Fig. 7e). As shown in Fig. 7f, p-STAT3^Y705^, p-AKT^S473^ and p-ERK1/2^T202/Y204^ in the tumor tissue sample in bazedoxifene- and combination-treated group was reduced.

## Discussion

IL11/GP130 signaling plays a critical role in tumorigenesis, tumor proliferation metastasis and chemoresistance in multiple types of cancers [12, 22, 26, 30, 31]. Both members of IL-6 family, IL-6 and IL-11, can act on the cells by similar interaction with receptor GP130 and lead to the intracellular signal. However, IL-11, rather than IL-6, plays a more prominent role in promoting colon cancer cell growth [22]. IL-11, a 19-kDa soluble factor first identified in bone marrow-derived stromal cells, is a member of GP130 cytokines that utilizes the GP130 signaling pathway shared by other cytokines of the same family [32]. Physiologically, IL-11 signaling plays an important role in thrombopoiesis, embryogenesis, cardiovascular fibrosis, immunomodulation, mucosal protection, hematopoiesis and promotion of stem cell development [16, 33]. The αreceptor subunits of IL-11, IL-11Rα, are often used to identify the expression pattern of IL-11 [34]. High IL-11 expression was reported to be associated with poor differentiation, larger tumor size, lymph node metastasis and inferior overall survival of colorectal cancer patients [35]. Its role in mediating cancer development is mainly through the activation of the JAK-STAT3 signaling pathway [16]. Persistent STAT3 activation has been identified to be a prominent feature in many cancers of epithelial origins. IL-11 stimulation hence results in a more epithelial-specific response. IL-11 signaling is a very important and novel potential therapeutic target for the treatment of gastrointestinal cancers, including colon cancers. However, only a few studies on targeting IL-11 or its receptor-αin cancers in pre-clinical models have been published so far [22, 36, 37]. In one study, administration of IL-11 signaling antagonist IL-11-Mutein reduced inflammation-associated colorectal cancer and gastric carcinoma in a mouse model [22]. After we identified the activation of GP130, IL-11, IL-11Rα and STAT3 expression in human colon cancer cells, we confirmed that the neutralized GP130 antibody could reduce the viability of human colon cancer cells. This provided the evidence that colon cancer may be treated by targeting IL-11. The computational simulation analysis of the bazedoxifene molecular structure has found that it can bind the D1 domain of GP130 and block IL-11, thus inhibiting IL-11/GP130 signaling and further preventing hexamer formation and the signaling cascade downstream of STAT3. Therefore, we selected bazedoxifene, which can directly target IL-11 in a computational model and has inhibitory effects on colon cancer. To exclude the possibility that bazedoxifene may exert its effect through the estrogen receptor, the expression of the latter was also examined, but no ER-α was detected in three colon cancer cells. We have demonstrated for the first time that the FDA-approved drug bazedoxifene can inhibit human colon cancer cells in vitro and in vivo by targeting IL-11/GP-130 signaling. The detailed mechanism involves inhibition of p-STAT3 and its nuclear translocation induced by IL-11 and, hence, inhibition of STAT3 downstream targets AKT, and ERK1/2.

In addition, we discussed the synergistic effect of bazedoxifene and oxaliplatin. In clinical practice, the chemoresistance to oxaliplatin limits its effectiveness. Although several studies investigated the mechanism for oxaliplatin resistance, including DNA hypermethylation, histone post-translational modifications and microRNAs [38–41], the role of IL-11 in oxaliplatin chemoresistance has not been discussed. The IL-11/GP130/STAT3 pathway is involved in drug resistance in a variety of human cancers, including colon cancer. Our results showed that elevated IL-11 can reduce the efficacy of oxaliplatin and induce the resistance to oxaliplatin in colon cancer. Clinically, in relapsed patients treated with 5-fluorouracil and leucovorin, the addition of oxaliplatin only gives rise to an estimated increase of two months in median survival time [42]. Unfortunately, most current treatment strategies including FOLFOX, have not been able to significantly increase the overall survival in colon cancer, creating a need for new combined therapies. We combined bazedoxifene with oxaliplatin to suppress the elevated IL-11, and the combination of bazedoxifene and oxaliplatin worked synergistically to inhibit colon cancer cell viability. The underlying mechanism involves blocking IL-11 signaling, which may increase the sensitivity to oxaliplatin. The cell migration and cell colony formation assays provided additional evidence that bazedoxifene increased the sensitivity of colon cancer to oxaliplatin. This not only explains a possible mechanism of oxaliplatin resistance acquired due to the IL-11 signaling but also provides a potential target in colon cancer prevention and therapy.

Bazedoxifene is an FDA-approved drug with an approximate 6% oral bioavailability. In healthy postmenopausal women, this drug has long been proved to be safe and well-tolerated in the range of doses [43]. Pharmacokinetic evaluations in healthy postmenopausal women found that bazedoxifene displayed linear pharmacokinetics with doses ranging from 5 to 40 mg, with no unexpected accumulation [43]. According to the literature calculation method [44], the estimated human equivalent dose converted from mice in our study is 0.8 mg/kg. The estimated total dose for patients with 50 kg body weight should be 40 mg, and this dose should be safe and tolerated.

## Conclusions

In conclusion, our results indicate that FDA-approved bazedoxifene, as a novel GP130 inhibitor that blocks IL-11 signaling and can be repurposed for the treatment of colon cancer. Combined bazedoxifene and oxaliplatin therapy may be a viable therapeutic approach for human colon cancer. Clinical trials are needed to confirm the efficacy of colon cancer patients using bazedoxifene.

## Additional file

Additional file 1: Docking modeling of Bazedoxifene to GP130 receptor. GP130 D1 (PDB code: 1P9M) domain is shown in grey Ribbon; bazedoxifene is rendered in green stick; IL-11 Trp168 and Leu72 are shown in red lines. Picture is made using AutoDockTools (ADT). (TIF 2907 kb)

## Abbreviations

AKT: protein kinase B
BrdU: Bromodeoxyuridine
ERK: Extracellular regulated protein kinases
GP130: Glycoprotein 130
SDS-PAGE: Sodium dodecyl sulfate polyacrylamide gel electrophoresis
STAT3: Signal transducer and activator of transcription 3
