# Correction to: Bazedoxifene as a novel GP130 inhibitor for Colon Cancer therapy

**DOI:** 10.1186/s13046-019-1381-y

**Published:** 2019-08-23

**Authors:** Jia Wei, Ling Ma, Yi-Hui Lai, Ruijie Zhang, Huameng Li, Chenglong Li, Jiayuh Lin

**Affiliations:** 1 Department of Hematology, Tongji Hospital, Tongji Medical College, Huazhong University of Science and Technology, Wuhan, 430030 People’s Republic of China; 2 Department of Biochemistry and Molecular Biology, University of Maryland School of Medicine, 108 N. Greene Street, Baltimore, MD 21201 USA; 3 33 Linsen Road, Chungshan District, Taipei, Taiwan; 4 Biophysics Graduate Program, The Ohio State University, Columbus, OH 43210 USA; 5 College of Pharmacy, University of Florida, Gainesville, FL 32610 USA

**Correction to: J Exp Clin Cancer Res**

**https://doi.org/10.1186/s13046-019-1072-8**

In the original publication of this article [1], there is an error in Fig. 4A.

The corrected Fig. 4A should be:

**Fig. 4:**
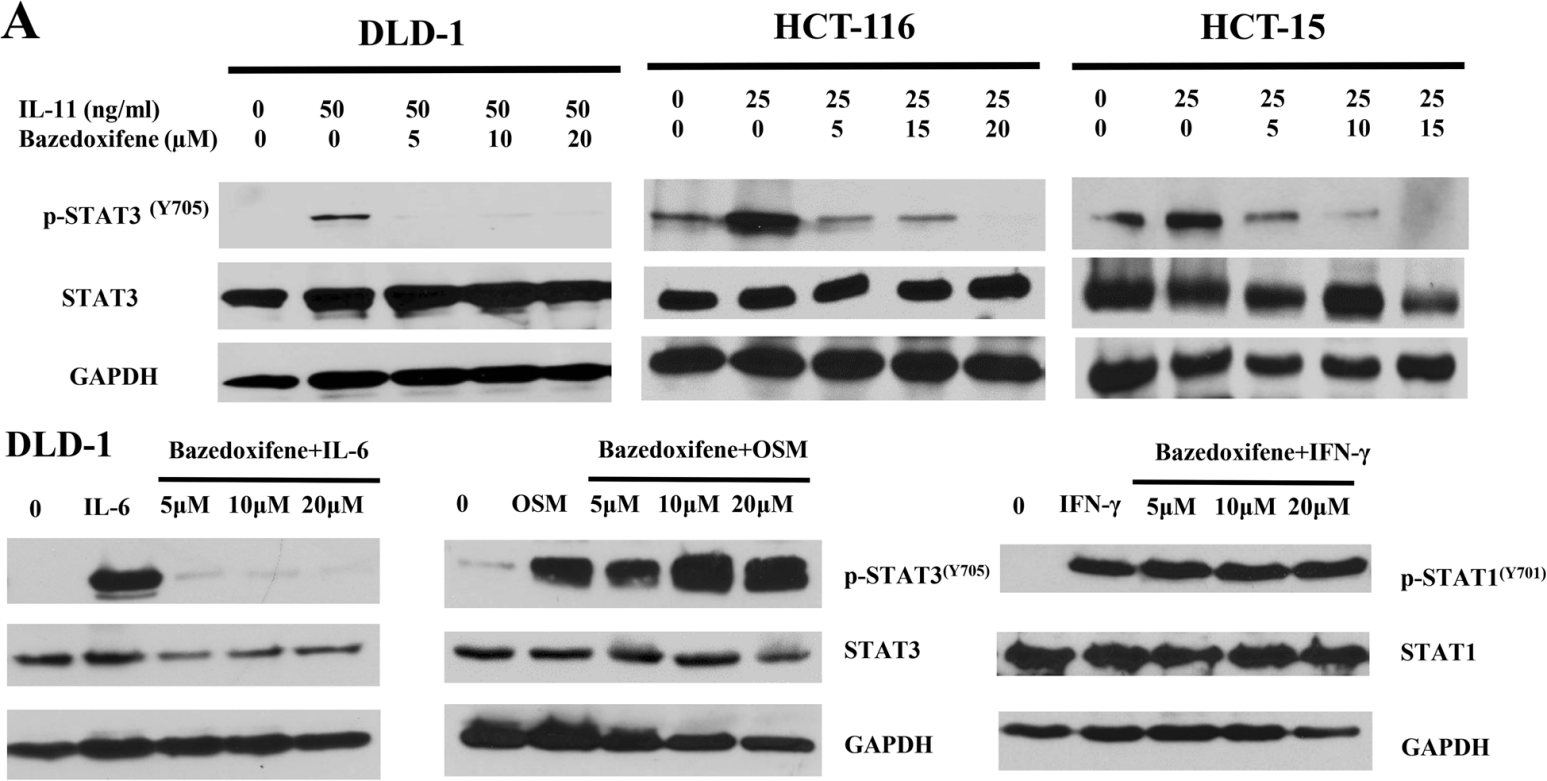
Bazedoxifene inhibits induction of STAT3 phosphorylation and cell proliferation by IL-11. **a**: DLD-1, HCT-116, and HCT-15 cells were starved in serum-free medium for 24 h and pre-treated with bazedoxifene (5~20 μM) for 2 h. Then, 50 ng/ml (DLD-1 cells) or 25 ng/ml IL-11 (HCT-116 and HCT-15 cells), 50 ng/ml OSM (DLD-1 cells) and 50 ng/ml IFN-γ (DLD-1 cells) were added for stimulation. The p-STAT3Y705, p-STAT1Y701, STAT3, STAT1 and GAPDH were assessed by western blot analysis.
