# Supplementary figures and images for: Bazedoxifene as a novel GP130 inhibitor for Colon Cancer therapy
Source: J Exp Clin Cancer Res. 2019 Feb 8;38:63. doi: 10.1186/s13046-019-1072-8 (PMC6368818; doi:10.1186/s13046-019-1072-8)

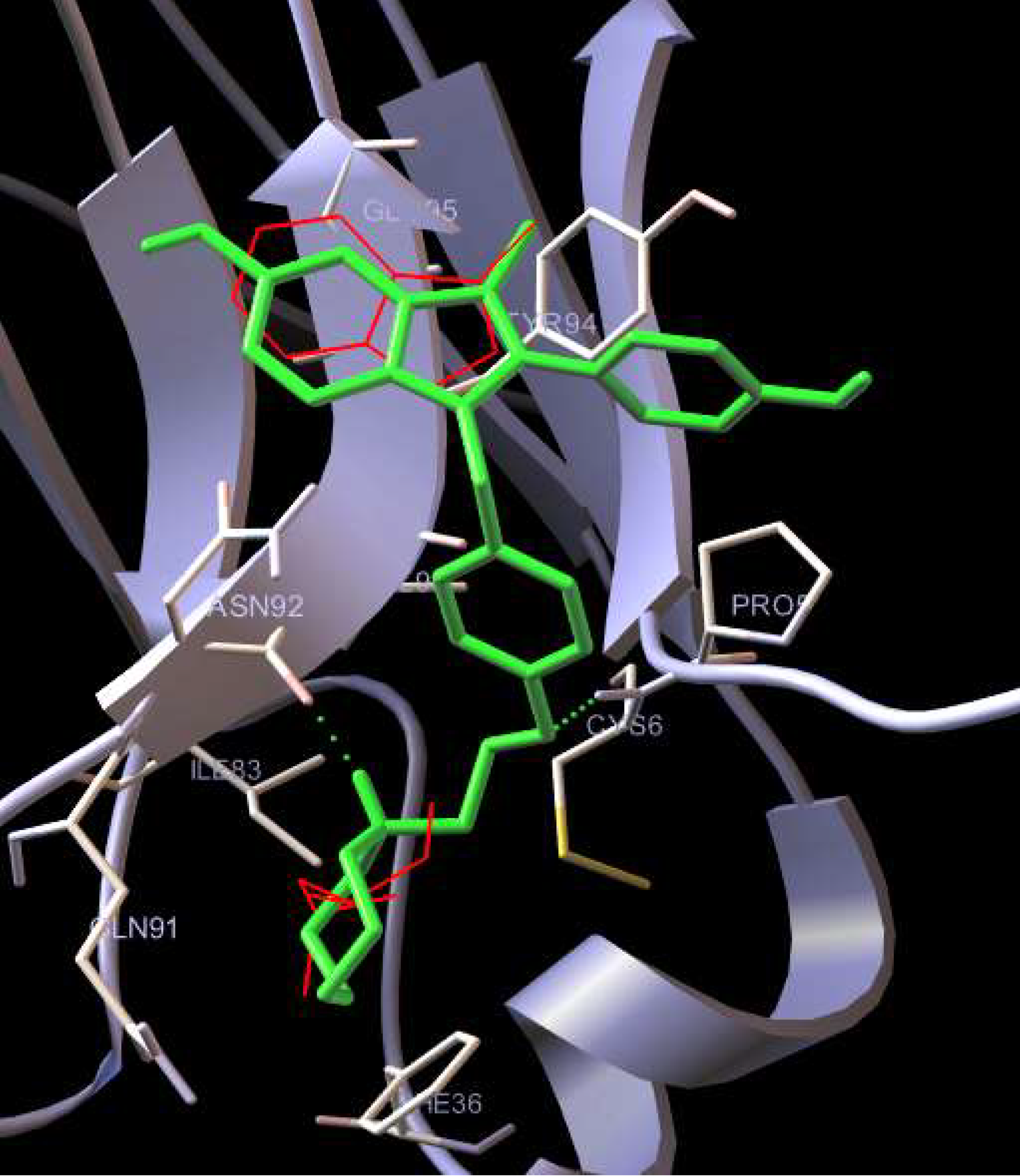

Supplement: Supplementary file 1 — Docking modeling of Bazedoxifene to GP130 receptor. GP130 D1 (PDB code: 1P9M) domain is shown in grey Ribbon; bazedoxifene is rendered in green stick; IL-11 Trp168 and Leu72 are shown in red lines. Picture is made using AutoDockTools (ADT). (TIF 2907 kb) [file 13046_2019_1072_MOESM1_ESM.tif]
